# Effects of caffeine supplementation on exercise-induced muscle damage: A systematic review

**DOI:** 10.1097/MD.0000000000051196

**Published:** 2026-10-30

**Authors:** Süleyman Mercan, Armağan Şahin Kafkas, Ilgın Ali Coşkun, Muhammed Emin Kafkas

**Affiliations:** a Faculty of Sport Sciences, İnönü University, Malatya, Türkiye.

**Keywords:** Caffeine supplementation, exercise physiology, muscle recovery, sports nutrition

## Abstract

**Background:**

Exercise-induced muscle damage (EIMD) commonly occurs following unaccustomed or high-intensity exercise and is characterized by delayed-onset muscle soreness, elevated muscle damage biomarkers, and transient declines in muscle function. Caffeine, a widely consumed ergogenic aid, has been proposed to mitigate these adverse effects; however, the evidence across studies remains equivocal. This systematic review aimed to synthesize findings from randomized controlled trials (RCTs) that investigated the effects of caffeine supplementation on markers of EIMD.

**Methods:**

Following preferred reporting items for systematic reviews and meta-analyses guidelines, a comprehensive search was conducted in PubMed, Scopus, and Google Scholar databases, supplemented by citation tracking. RCTs published between 2005 and 2025 that included human participants and reported direct or indirect indicators of EIMD were eligible. Fifteen RCTs (n = 222) were included and assessed for methodological quality using the PEDro scale.

**Results:**

Caffeine doses ranged from 3 to 6 mg/kg. Among the 6 studies assessing delayed-onset muscle soreness, 4 reported significant reductions in perceived soreness within 24 to 48 hours post-exercise, whereas 2 found no effect or adverse responses. Eleven studies examined biochemical markers; most detected no significant differences between caffeine and placebo, while 2 observed higher creatine kinase levels in the caffeine group. Five studies evaluated functional performance; some indicated attenuation of decrements in maximal voluntary isometric contraction or isokinetic torque, although results were inconsistent.

**Conclusion:**

Caffeine supplementation may transiently reduce subjective muscle soreness and, under certain conditions, help preserve muscle function following EIMD, but its effects on biochemical markers are inconsistent. The overall efficacy appears to depend on factors such as exercise modality, dosage, timing, and inter-individual variability. Larger, well-controlled RCTs with standardized protocols are needed to clarify caffeine’s role in muscle damage and recovery.

## 1. Introduction

In recent years, research on the role of dietary supplements and nutritional strategies in enhancing athletic performance and optimizing post-exercise recovery has gained increasing attention. Among these, caffeine supplementation has emerged as a particularly relevant topic because of its potential to modulate exercise-induced muscle damage (EIMD) and support recovery processes. Caffeine, a widely consumed ergogenic aid, is naturally present in >60 plant species and is commonly ingested in beverages such as coffee, tea, maté, guarana, and cola. Chemically, caffeine (1,3,7-trimethylxanthine) belongs to the methylxanthine family and exerts multiple physiological effects via central and peripheral mechanisms.^[1–3]^

### 1.1. Physiological and molecular mechanisms of caffeine

Caffeine increases the release of catecholamines (dopamine, adrenaline, and noradrenaline), thereby enhancing alertness and neuromuscular activation.^[4–6]^ This heightened sympathetic activity may also contribute to delayed glycogen depletion and enhanced fat oxidation, thereby sparing muscle glycogen during exercise.^[7,8]^ However, findings remain inconsistent, with variability attributed to dose, training status, habitual caffeine use, feeding state, and exercise intensity.^[9–11]^

Caffeine intake increases circulating catecholamine levels, which in turn enhance lipolysis and promote the mobilization of free fatty acids, thereby increasing the availability of fat as a substrate for active muscles.^[12,13]^ This mechanism has been hypothesized to contribute to glycogen sparing during exercise by shifting substrate utilization toward fat oxidation.^[14]^ However, findings remain inconsistent, and no consensus has been reached on whether caffeine directly preserves muscle glycogen stores.^[15,16]^ Variability across studies may stem from differences in training status, habitual caffeine consumption, dosage, feeding or fasting conditions, and exercise intensity.^[17,18]^ Collectively, these factors may modulate the metabolic response to caffeine and partially explain the heterogeneity observed in the exercise outcomes.

Caffeine enhances calcium release from the sarcoplasmic reticulum via ryanodine receptors,^[19]^ which may increase muscle contractility and delay fatigue.^[20,21]^ However, these effects are dose-dependent; moderate doses may improve excitation–contraction coupling, and excessive intake may cause membrane depolarization, dehydration, or gastrointestinal distress.^[4,22]^ Caffeine may also exert neurostimulatory and anti-inflammatory effects by modulating neurotransmission and immune signaling. It can inhibit γ-aminobutyric acid type A receptor activity, reduce inhibitory tone, and promote alertness.^[23]^ In addition, caffeine intake has been associated with decreased levels of pro-inflammatory markers, such as C-reactive protein, interleukin (IL)-1β, IL-6, and tumor necrosis factor-α, and increased levels of anti-inflammatory mediators, such as IL-10 and adiponectin,^[24–26]^ which may contribute to improved recovery and reduced secondary muscle damage following exercise.

One of the primary mechanisms by which caffeine exerts its physiological effects is adenosine receptor antagonism. Adenosine normally binds to its receptors (A_1_, A_2_A, A_2_B, and A_3_) located throughout the central nervous system and peripheral tissues, exerting inhibitory effects, such as neuronal hyperpolarization, reduced excitatory neurotransmitter release, and decreased heart rate, all of which promote relaxation and fatigue.^[27,28]^ Due to its structural similarity to adenosine, caffeine competitively blocks these receptors, particularly the A_1_ and A_2_A subtypes, thereby preventing adenosine-mediated inhibition and resulting in increased neuronal firing, elevated catecholamine release, and heightened alertness.^[29,30]^

In the context of exercise, this mechanism may contribute to enhanced arousal, reduced perception of effort, and attenuation of central fatigue, indirectly supporting performance and recovery following muscle-damaging activity.^[31,32]^ Moreover, adenosine receptor blockade has been associated with modulation of pain perception and anti-inflammatory signaling, which could play a complementary role in mitigating the symptoms of EIMD.

### 1.2. Relationship between delayed-onset muscle soreness (DOMS) and EIMD

DOMS is a transient physiological response that typically arises 8 to 24 hours after unaccustomed or high-intensity exercise, especially those involving eccentric contractions, and peaks between 24 and 72 hours before resolving within 3 to 5 days.^[33,34]^ It is characterized by localized discomfort, stiffness, swelling, and temporary loss of muscle function, leading to impairments in strength, power, flexibility, and movement efficiency.^[35–37]^ Unlike acute exercise-induced pain, which occurs during activity and subsides within hours, DOMS reflects a delayed inflammatory response associated with structural muscle disruptions.^[38,39]^

The pathophysiology of DOMS has been attributed to multiple interrelated mechanisms, including mechanical microtrauma, connective tissue disruption, oxidative stress, inflammatory signaling, and alterations in calcium homeostasis.^[40,41]^ Among these, EIMD is considered the primary trigger.^[42,43]^ Microscopic tears in myofibrils and associated structural proteins (e.g., titin, dystrophin, actin, and myosin) initiate a cascade of cellular responses, leading to the release of intracellular enzymes, such as creatine kinase (CK), lactate dehydrogenase (LDH), and myoglobin(Mb), into the bloodstream.^[44,45]^

The ensuing inflammatory phase is marked by neutrophil and macrophage infiltration, which contributes to secondary muscle damage through the generation of reactive oxygen species and pro-inflammatory cytokines.^[46]^ Despite this transient damage, the inflammatory response also promotes tissue remodeling and adaptation, facilitating subsequent muscle regeneration and increasing resilience to future exercise stimuli.^[47]^

Collectively, DOMS can be viewed as a clinical manifestation of EIMD, reflecting both structural disruption and inflammatory repair processes rather than a purely metabolic phenomenon. Understanding this relationship is essential for evaluating the potential modulatory effects of interventions such as caffeine, which may influence pain perception, inflammatory signaling, and functional recovery following muscle-damaging exercise.

### 1.3. The relationship between caffeine and muscle damage

EIMD is a common physiological phenomenon experienced by individuals engaging in unaccustomed or high-intensity training aimed at improving their health or athletic performance.^[40,48]^ Consequently, developing effective and rapid recovery strategies has become a major focus in sports and exercise science. Among these strategies, caffeine supplementation has long attracted research attention because of its potential to mitigate muscle damage and support recovery processes.^[41,49,50]^

Evidence from several randomized controlled trials (RCTs) suggests that moderate caffeine intake may alleviate markers of muscle damage, particularly by reducing DOMS, improving perceived exertion, and preserving muscle function.^[51–56]^ However, the findings in the literature remain inconsistent. Some studies have reported no significant impact of caffeine on biochemical markers (e.g., CK and Mb) or recovery rates,^[57–59]^ while others have even observed potential adverse effects, such as increased muscle damage markers or delayed regeneration.^[60,61]^

These discrepancies may be attributed to methodological differences, including variations in exercise modality, caffeine dosage, ingestion timing, participants’ habitual caffeine use, and individual sensitivity. As a result, the effect of caffeine on EIMD remains controversial. Therefore, understanding whether caffeine exerts a protective, neutral, or detrimental effect under specific conditions is of both theoretical and practical relevance.

Accordingly, the present systematic review aimed to synthesize evidence from RCTs to evaluate the effects of caffeine supplementation on EIMD and to identify potential moderating factors influencing its efficacy.

## 2. Materials and methods

### 2.1. Study design

This systematic review was conducted in accordance with the preferred reporting items for systematic reviews and meta-analyses (PRISMA 2020) guidelines.^[62]^ The guiding research question was: *Does caffeine supplementation influence EIMD ?*

### 2.2. Search strategy

This review was structured according to the PICOS framework to ensure a systematic and transparent selection process. The Population (P) comprised healthy adults who performed exercise protocols designed to induce muscle damage. The Intervention (I) included caffeine supplementation at any dosage, form, or timing, whereas Comparison (C) involved placebo or control conditions without caffeine intake. The Outcomes (O) focused on both direct (e.g., magnetic resonance imaging and muscle biopsy) and indirect (e.g., DOMS, CK, LDH, Mb levels, and performance loss) indicators of muscle damage. Finally, the Study design (S) was limited to RCTs, which provided the highest level of evidence for causal inference.

During the search process, the Boolean operator rules of each database were examined, and keywords were used. A comprehensive search strategy was implemented using a combination of controlled vocabulary and free text terms related to caffeine supplementation and EIMD. The search terms included variations and synonyms of “caffeine,” “coffee,” “caffeine supplementation,” “delayed onset muscle soreness,” “DOMS,” “exercise-induced muscle damage,” “EIMD,” “muscle recovery,” “muscle repair,” and “muscle inflammation.” Boolean operators (AND/OR) were applied as appropriate to maximize the sensitivity and specificity across databases. Electronic searches were conducted in PubMed, Scopus, and Google Scholar, covering the period January 2005 to October 2025.

#### 2.2.1. Inclusion criteria

Studies were eligible for inclusion if they were RCTs conducted in human participants and investigated EIMD. Studies published between 2005 and 2025 were included. Both direct measurement methods (e.g., magnetic resonance imaging and muscle biopsy) and indirect indicators (e.g., biochemical markers such as CK or LDH, DOMS, muscle swelling, reductions in performance, or range-of-motion limitations) were considered acceptable outcome measures.

#### 2.2.2. Exclusion criteria

Studies were excluded if they included animal models that combined caffeine with other nutritional supplements or interventions; focused on genetic polymorphisms or genotype-specific responses; were non-original publications such as reviews, theses, letters to the editor, or case reports; or were not published in English.

### 2.3. Study selection

All the selection stages were independently screened and reviewed by 2 authors, and any disagreements during the evaluation process were resolved through discussion and consensus. This study adhered to the PRISMA 2020 guidelines and was reported using the updated PRISMA flow diagram^[62]^ (Fig. 1).

**Figure 1.**
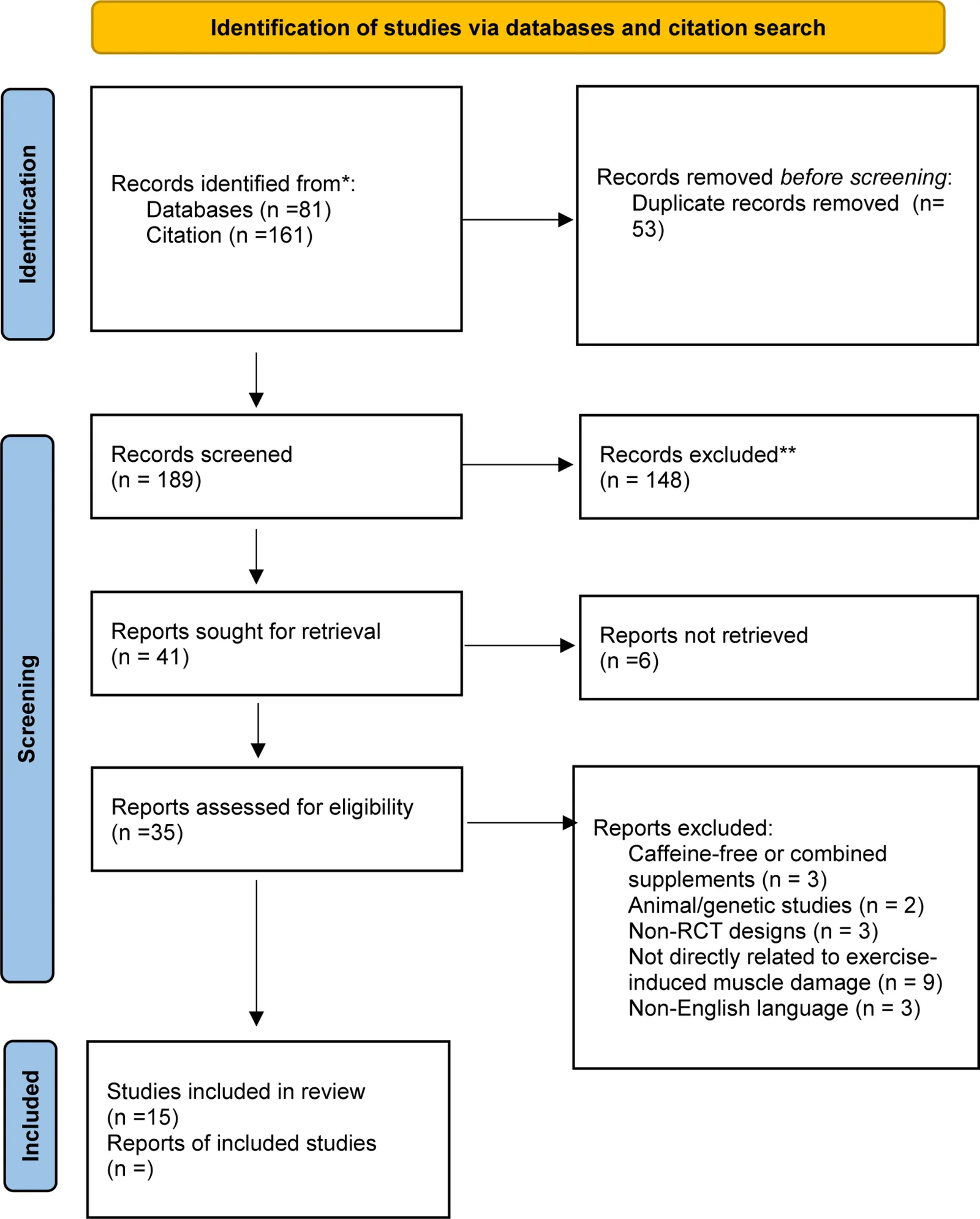
PRISMA flow diagram of the study selection process. We identified 242 records, comprising 81 from database searches and 161 from citation searching. After removing 53 duplicates, we screened 189 records, of which 148 were excluded. We sought 41 reports for retrieval, but 6 could not be retrieved. We assessed 35 reports for eligibility and excluded 20. Ultimately, 15 studies were included in the review. PRISMA = preferred reporting items for systematic reviews and meta-analyses.

#### 2.3.1. First phase: database search

In the 1st phase, all records identified through database searches were electronically compiled, and duplicate entries were removed. The remaining studies were screened by title and abstract, and those that met the predefined inclusion criteria were selected for full-text evaluation. Full-text articles were retrieved whenever possible, and unavailable records were documented. Each full-text article was assessed in detail for eligibility, and studies that did not meet the inclusion criteria were excluded with reasons recorded.

#### 2.3.2. Second phase: citation search

In the 2nd phase, a citation-tracking search was performed using the reference lists of the studies included in the 1st phase. Newly identified records were merged with the initial dataset, and duplicate studies were removed. Titles, abstracts, and full texts were evaluated using the same inclusion and exclusion criteria as applied in the 1st phase. Any studies excluded at this stage were documented along with their rationale. The remaining eligible studies were included in the final qualitative synthesis and reported in accordance with the PRISMA 2020 flow diagram.^[62]^

### 2.4. Data extraction and quality assessment

All eligible studies were systematically documented by reviewers using a predefined structured data extraction form to ensure accuracy and consistency. The extracted data included the 1st author and publication year, country, study design, participant sex and training status, applied exercise protocol, caffeine dosage, timing of administration, and indicators of muscle damage. The key characteristics of the included studies are summarized in Table 1.

**Table 1:** Characteristics of the included studies.

Author (year)	Country	RCT	N (M/F)	Training status	Exercise protocol	Caffeine dose (mg/kg)	Timing	Washout period	Muscle damage indicators
Green et al (2018)^[65]^	USA	Crossover, double blind, and placebo controlled	16 (8/8)	Physically active individuals	Leg extension 10 × 10	6 mg/kg	30 min before exercise	1 wk	Maximal isometric torque, maximal isokinetic torque, fatigue index, and VAS
Hurley et al (2013)^[53]^	USA	Crossover, double blind, and placebo controlled	9 (9/0)	Recreational-level resistance training participants	4 × 10 biceps curl and 5 sets max rep	5 mg/kg	1 h before exercise	1 wk	CK, VAS, BORG CR10, and MAX REP
Chen et al (2019)^[52]^	Taiwan	Crossover, double blind, and placebo controlled	20 (10/10)	Elite university students (tennis, basketball, English football)	30 min %70 VO_2_max downhill running %15 incline	6 mg/kg	Caffeine or placebo was administered 24 and 48 h after exercise.	Not specified	CK, VAS, serum potassium (K^+^), glucose, and lactate levels
Al-Nawaiseh et al (2020)^[60]^	Jordan and USA	Crossover, double blind, and placebo controlled	11 (/9/2)	Regional-level runners and triathletes	30 min %70 VO_2_max downhill running %10 incline	5 mg/kg	1 h before the 5 km performance test, 48 h after muscle damage	3 wk	VAS, force algometer, BORG RPE, HR, and 5 km time trial
Mahdavi et al (2012)^[72]^	Iran	Crossover, double blind, and placebo controlled	26 (0/26)	Basketball players	A 30-s Wingate test was used.	5 mg/kg	Administered 70 min before exercise	1 wk	CK
Caldwell et al (2017)^[51]^	USA	Parallel, double blind, and placebo controlled	30 (25/5)	Cyclists	A 164 km endurance cycling event was used.	3 mg/kg	Administered after exercise and on the following days	None	RPMS and LEF
Maridakis et al (2007)^[54]^	USA	Double-blind, placebo-controlled, and repeated-measures design	9 (0/9)	University students	Performed 8 × 8 eccentric movements using electrical stimulation.	5 mg/kg	Administered 24 and 48 h after exercise	None	MVIC and VAS
Machado et al (2008)^[66]^	Brazil	Crossover, double blind, and placebo controlled	10 (10/0)	At least 6 mo of resistance training	Bench press, leg press, leg extension, lat pulldown, and biceps curl exercises (3 sets × 10 repetitions, 75% 1RM, 2-min rest).	5 mg/kg	Caffeine or placebo intake 1 h before exercise	2 wk	CK, LDH, and VAS
Pereira et al (2012)^[70]^	Brazil	Parallel, double-blind, and placebo-controlled	20 (20/0)	Professional football players	12 set × 10 sprint (20 m) + Yo-Yo IRT	5.5 mg/kg	45 min before exercise	None	Muscle pain (subjektif) and CK (0, 24, and 48 h)
Ribeiro et al (2016)^[71]^	Brazil	Crossover, double blind, and placebo controlled	6 (6/0)	Elite handball players	4 sets × 30-s continuous vertical jumps (VJ), 60-s rest.	6 mg/kg	1 h before exercise	1 wk	CK and decrease in muscle strength
Machado et al (2009)^[58]^	Brazil	Parallel, double blind, and placebo controlled	20 (20/0)	Trained football players	6 sets × 10 × 20 m sprints, 10-s rest, 5-min walking between sets.	4.5 mg/kg	45 min before exercise	None	CK, LDH, AST, ALT, AP, and γ-GT
AbuMohd (2023)^[67]^	Jordan	Crossover, double blind, and placebo controlled	11 (11/0)	Men with at least 5 yr of resistance training	3 sets × repetitions to failure (bench press, biceps curl, shoulder press, leg press, back squat, leg extension), 70% 1-RM, 90-s rest.	5 mg/kg	1 h before exercise	1 wk	CK, CK-MB, and Mb
AbuMoh’d et al (2020)^[69]^	Jordan	Crossover, double blind, and placebo controlled	15 (15/0)	Long-distance runners	5 resistance exercises (60% 1-RM, 3 × 10) + incremental running test	6 mg/kg	1 h before exercise	1 wk	CK, LDH, CK-MB, and Mb
Gharaat et al (2020)^[73]^	Iran	Crossover, double blind, and placebo controlled	9 (9/0)	Elite male rowers	2000 m rowing ergometer test (2 trials, 6.5 h apart)	3 and 6 mg/kg	30 min before the test	6 d	Serum CK, blood lactate, performance loss
Filip-Stachnik et al (2023)^[68]^	Poland	Crossover, double blind, and placebo controlled	10 (10/0)	Men accustomed to resistance training	7 exercises, 3 sets 70% 1RM, to failure, 4-s eccentric, 3-min rest	3 mg/kg	1 h before exercise	1 wk	Serum CK, LDH, perceived pain, performance

ALT = alanine aminotransferase, AP = alkaline phosphatase, AST = aspartate aminotransferase, BORG CR 10 = Borg category-ratio 10 scale, BORG RPE = Borg rating of perceived exertion, CK = creatine kinase, CK-MB = creatine kinase–myocardial band, HR = heart rate, LDH = lactate dehydrogenase, LEF = lower extremity functionality, MAX REP = maximum repetitions, Mb = myoglobin, MVIC = maximal voluntary isometric contraction, RCT = randomized controlled trial, RPMS = rating of perceived muscle soreness, VAS = visual analogue scale, γ-GT = gamma-glutamyl transferase.

Methodological quality was evaluated using the physiotherapy evidence database (PEDro) scale,^[63]^ a validated and reliable tool designed to assess the quality of RCTs.^[64]^ The scale comprises 11 items, of which 10 are scored, with each satisfied criterion receiving a score of 1 and the unmet criteria scored as 0, resulting in a total score ranging from 0 to 10. Two independent reviewers performed the assessments, and any discrepancies were resolved through consensus. The PEDro scores of the included studies are shown in Table 2.

**Table 2:** Methodological quality assessment of the included studies PEDro scale.

Criteria												
Yazar	1*	2	3	4	5	6	7	8	9	10	11	PEDro score
Green et al^[65]^	1	1	1	1	1	1	1	1	1	1	1	10
Hurley et al^[53]^	0	1	1	1	1	1	1	1	1	1	1	10
Chen et al^[52]^	1	1	1	1	1	1	1	1	1	1	1	10
Al-Nawaiseh et al^[60]^	1	1	0	1	1	0	1	1	0	1	1	7
Mahdavi et al^[72]^	0	1	1	1	1	1	1	1	1	1	1	10
Caldwell et al^[51]^	1	1	1	1	1	1	1	1	1	1	1	10
Maridakis et al^[54]^	1	1	1	0	1	1	1	1	1	1	1	9
Machado et al^[66]^	1	1	0	1	1	0	0	1	0	1	1	6
Pereira et al^[70]^	1	1	0	1	1	0	0	1	1	1	1	7
Ribeiro et al^[71]^	1	1	1	1	1	0	1	0	1	1	1	8
AbuMohd^[67]^	1	1	1	1	1	0	1	0	1	1	1	8
AbuMohd et al^[69]^	1	1	1	1	1	0	1	1	1	1	1	9
Gharaat et al^[73]^	1	1	1	1	1	0	1	1	1	1	1	9
Filip-Stachnik et al^[68]^	1	1	1	1	1	1	1	1	0	1	1	9
Machado et al^[58]^	1	1	1	1	1	0	1	0	1	1	1	8

* Criterion 1: Not scored because it relates to eligibility criteria and external validity; therefore, it does not reflect the quality dimensions.

PEDro = physiotherapy evidence database.

## 3. Results

### 3.1. Screening and selection process of studies

In the 1st phase, 81 studies were identified through database searches conducted in PubMed, Scopus, and Google Scholar. After removing 11 duplicate records, 70 studies remained for title and abstract screenings. During this stage, 52 studies were excluded as they did not meet the predefined inclusion criteria. The full texts of 18 potentially eligible studies were retrieved; however, 4 could not be accessed. The remaining 14 studies underwent detailed full-text assessment, and 6 were excluded for documented reasons. Consequently, 8 studies were included in the qualitative synthesis after the 1st phase.

In the 2nd phase, forward citation searches were performed based on 8 studies that were initially included. This search identified an additional 161 records, which were merged with the previously obtained dataset, resulting in 242 records. After removing 53 duplicates, 189 studies were screened at the title and abstract levels. Of these, 148 were excluded as they did not meet the eligibility criteria. The full texts of the remaining 41 studies were sought, but 6 were unavailable. After a detailed evaluation of the 35 accessible full texts, 20 were excluded for several reasons. Ultimately, 15 studies met all the criteria and were included in the final qualitative synthesis of this systematic review (Fig. 1).

### 3.2. General characteristics of the included studies

Fifteen RCTs met the inclusion criteria and were included in this systematic review. The key characteristics of these studies are summarized in Table 1. The studies were published between 2007 and 2023 and conducted across 6 countries: the United States, Taiwan, Jordan, Iran, Brazil, and Poland.

The sample sizes ranged from 6 to 30 participants, yielding a total of 222 participants. Of these, 162 (72.97%) were male and 60 (27.03%) were female. Some studies included only 1 sex, whereas others included mixed cohorts. Regarding training background, 157 participants (70.7%) were professional or amateur athletes, 40 (18.0%) had resistance training experience, 16 (7.2%) were physically active, and 9 (4.0%) were university students.

Various exercise protocols have been used to induce muscle damage. These included isokinetic contractions,^[65]^ electrical stimulation,^[54]^ and resistance exercises.^[53,66–69]^ Other protocols involved sprint-based tasks,^[58,70,71]^ downhill running,^[52,60]^ the Wingate anaerobic test,^[72]^ a 2000 m rowing ergometer test,^[73]^ and a 164 km cycling trial.^[51]^

Across the studies, the caffeine doses ranged from 3.0 to 6.0 mg/kg. The most common dose was 5 mg/kg (6 studies), followed by 6 mg/kg (4 studies), and 3 mg/kg (2 studies). The remaining studies administered 4.5 or 5.5 mg/kg, while 1 trial compared 3 and 6 mg/kg. Caffeine was typically administered 30 to 70 minutes before exercise in 11 studies (73.3%), whereas 3 studies (20%) provided caffeine 24 to 48 hours post-exercise, and 1 study (6.7%) administered caffeine 48 hours after exercise before a subsequent test.

Habitual caffeine consumption was quantitatively reported in 11 studies, qualitatively in 3, and not assessed in 2. Using the 3 mg/kg/d threshold,^[74]^ participants were categorized as low/light (<3 mg/kg/d) or moderate/high (>3 mg/kg/d). In 9 of the 11 quantitative studies, 128 participants (57.7%) were low/light consumers, while 1 study^[51]^ reported 30 participants (13.5%) as moderate/high consumers.

Among the 3 studies with qualitative data, 35 participants (15.8%) were described as “low/moderate habitual caffeine consumers,”^[68]^ “non-regular caffeine consumers,”^[73]^ or “consuming at least 1 caffeinated beverage 5 days/wk.”^[65]^ In the 2 studies that did not report caffeine consumption, 29 participants (13.1%) were included.

### 3.3. Methodological quality assessment

The methodological quality of the included studies was assessed using the PEDro scale (Table 2). Overall, the majority of the studies demonstrated moderate to high methodological quality.

Specifically, 5 studies (33.3%) achieved the maximum score (10/10), while an additional 4 studies (26.7%) scored 9/10, resulting in a total of 9 studies (60%) meeting high methodological standards (≥9/10). The remaining 6 studies received scores ranging from 6 to 8, indicating moderate quality.

The most frequent methodological shortcomings were related to the blinding and allocation procedures. In particular, participant blinding (criterion 6) was not achieved in 6 studies (40%), therapist or intervention blinding (criterion 7) in 3 studies (20%), and random allocation (criterion 3) in 4 studies (26.7%). These omissions may have introduced potential performance or selection biases, warranting a cautious interpretation of the results.

Regarding study design, 11 studies (73.3%) adopted a double-blind crossover design, incorporating washout periods of 6 days (n = 1), 7 days (n = 7), 14 days (n = 1), and 21 days (n = 1). Additionally, 3 studies (20%) used a double-blind parallel-group design, whereas 1 study (6.7%) employed a double-blind repeated-measures design (Table 1).

### 3.4. The effect of caffeine intake on markers of muscle damage

Fifteen studies examining the effects of caffeine supplementation on EIMD through subjective soreness, biochemical markers, and functional performance outcomes were included in this review.

#### 3.4.1. Delayed-onset muscle soreness (DOMS)

Six studies (40%) investigated the effects of caffeine on DOMS. Four studies reported the analgesic effects of caffeine. Hurley et al^[53]^ found significantly lower overall and palpation-induced pain at 48 hours post-exercise, although differences at 72 hours were not significant. Chen et al^[52]^ also observed a general reduction in DOMS scores, which was more pronounced in male participants. Similarly, Caldwell et al^[51]^ reported significantly lower pain scores during the 1st day, particularly at midday, but the effect diminished thereafter. Maridakis et al^[54]^ noted significant reductions in pain during maximal voluntary isometric contraction (MVIC), but not during submaximal eccentric exercise. In contrast, Green et al^[65]^ found no significant differences between caffeine and placebo groups at any time point, while Al-Nawaiseh et al^[60]^ observed higher visual analogue scale pain scores in the caffeine group.

#### 3.4.2. Biochemical markers of muscle damage

Eleven studies (73.3%) evaluated biochemical indicators, most frequently CK, LDH, aspartate aminotransferase (AST), alanine aminotransferase (ALT), Mb, and CK-myocardial band. Two studies reported significantly higher CK levels following caffeine supplementation compared to placebo.^[67,73]^ Notably, Gharaat et al^[73]^ found that CK levels increased in all groups post-exercise, with a greater increase in the low-dose caffeine group (3 mg/kg, +33.9%) than in the high-dose group (6 mg/kg, +14.1%). The remaining 9 studies found no significant differences between the caffeine and placebo conditions for CK or other markers (e.g., LDH, AST, and ALT). In most trials, enzyme levels increased due to exercise (time effect) but were unaffected by caffeine intake.

#### 3.4.3. Functional performance measures

Five studies assessed performance outcomes following EIMD. The results were heterogeneous across protocols and time points. Chen et al^[52]^ reported that caffeine significantly attenuated MVIC loss before and after fatigue. Maridakis et al^[54]^ observed a nonsignificant increase in MVIC. Green et al^[65]^ found that caffeine significantly improved isokinetic torque (+9.4%) 24 hours post-damage, but had no effect on isometric torque or fatigue index. Al-Nawaiseh et al^[60]^ reported no significant differences in 5 km running performance, heart rate, or perceived exertion at 48 hours. Caldwell et al^[51]^ observed a transient improvement in lower extremity function, which was significant only at midday on the 1st day.

## 4. Discussion

This systematic review aimed to comprehensively synthesize current evidence on the effects of caffeine supplementation on EIMD. Across the 15 RCTs included, caffeine was administered either before or after exercise protocols specifically designed to induce muscle damage, and at least 1 validated muscle damage marker was assessed. Given that EIMD is a multifactorial phenomenon, the review encompassed both direct (e.g., imaging and biopsy) and indirect (e.g., soreness, biochemical markers, and performance loss) indicators. However, none of the included studies employed direct assessment methods such as magnetic resonance imaging or muscle biopsy; instead, they relied on indirect measures including DOMS, enzyme activity (CK and LDH), and functional performance decrements.

### 4.1. Caffeine and muscle soreness

DOMS is the most common symptom of EIMD, typically arising after eccentric or unaccustomed contractions.^[35,75,76]^ Several meta-analyses and reviews have suggested that caffeine supplementation may attenuate DOMS,^[77–79]^ but the evidence remains mixed.

Among the 6 studies included in this review, 4 reported the analgesic effects of caffeine compared to placebo.^[51–54]^ In these trials, caffeine reduced soreness 24 to 48 hours post-exercise, particularly following eccentric or high-intensity resistance protocols. This benefit has been attributed to the antagonism of adenosine receptors by caffeine, which modulates central pain processing and dampens inflammatory responses.^[30,80]^ For instance, Hurley et al^[53]^ found reductions at 48 hours, while Maridakis et al^[54]^ and Chen et al^[52]^ reported effects at both 24 and 48 hours, the latter with a more pronounced effect in men. Caldwell et al^[51]^ observed reduced soreness only on the 1st day after a 164 km cycling protocol, suggesting that benefits may be short-lived in endurance exercise dominated by metabolic rather than mechanical stress.

In contrast, Green et al^[65]^ found no difference following eccentric isotonic leg extensions, and Al-Nawaiseh et al^[60]^ reported higher pain scores after downhill running with caffeine than with placebo. These contradictory outcomes highlight variability across exercise modes, populations, and study designs.

The dose also appears to influence outcomes. At 5 mg/kg, caffeine reduced DOMS in resistance-trained individuals^[53,54]^ but not in endurance-trained athletes.^[60]^ At 6 mg/kg, the effects ranged from^[52]^ to neutral.^[65]^ At 3 mg/kg, only Caldwell et al^[51]^ reported a modest early effect. These differences suggest that ergogenic and analgesic responses may depend on training status, sex, and exercise-induced stressors rather than the dose alone.

In summary, caffeine supplementation appeared to be most effective at attenuating DOMS after high-intensity eccentric or resistance protocols, particularly in recreationally trained populations. However, the findings are inconsistent in endurance contexts and across different dosing regimens, indicating that the hypoalgesic effects of caffeine are conditional and not universally reproducible.

### 4.2. Biochemical markers of muscle damage

EIMD is characterized by structural disruption of myofibrils, particularly Z-line misalignment, and impairment of excitation–contraction coupling.^[46,81]^ This initiates a cascade of secondary damage, driven by disturbed calcium homeostasis and elevated intracellular Ca^2+^, which activates proteolytic enzymes, such as calpains and phospholipase A_2_.^[82,83]^ The subsequent inflammatory response involves neutrophil infiltration, oxidative stress, and macrophage polarization from the M1 (pro-inflammatory) to M2 (anti-inflammatory) phenotypes, ultimately facilitating muscle repair and adaptation.^[46,84]^ Disruption of sarcolemmal integrity leads to the leakage of intracellular proteins into the bloodstream, with CK, LDH, AST, ALT, and Mb being the most widely used biochemical indicators.^[85]^ Among these, CK is the predominant marker; however, its validity as a surrogate for the magnitude of muscle damage or functional impairment remains controversial.^[86]^ Evidence suggests that CK levels are influenced by factors such as hydration status, exercise type, and individual variability, which limits their correlation with functional recovery.^[87,88]^

Across 11 studies assessing the impact of caffeine on biochemical markers, CK was universally measured, often accompanied by LDH, AST, ALT, or Mb. Only 2 studies^[67,73]^ reported significantly higher CK levels in caffeine groups compared to placebo, suggesting that caffeine’s stimulant and analgesic properties may permit greater exercise intensity and thus greater muscle stress.^[89]^ However, the inconsistency between doses, such as higher CK with 3 mg/kg than with 6 mg/kg, indicates that the response is not linear and may be modulated by dose, genetic polymorphisms, exercise modality, and hormonal milieu.^[90–92]^

The remaining 9 studies found no significant differences between the caffeine and placebo groups, although typical post-exercise increases in CK and other enzymes were observed, regardless of supplementation.^[52,53,58,66,68–72]^ This aligns with previous reviews reporting no consistent ergogenic or protective effects of caffeine on biochemical markers.^[79]^

In summary, the current evidence indicates that caffeine supplementation does not reliably attenuate biochemical markers of muscle damage. The observed heterogeneity likely reflects the complex interplay of dose–response relationships, genetic variability, and exercise characteristics. Future studies should adopt standardized protocols, integrate objective imaging with biochemical assays, and explore sex- and genotype-specific responses to clarify the role of caffeine in biochemical recovery following EIMD.

### 4.3. Assessment of physical performance loss due to muscle damage

In addition to biochemical markers and subjective measures, reduction in physical performance is considered a key indicator of EIMD. Among these, strength loss is widely recognized as a reliable and sensitive marker.^[88,93]^ This decline is primarily attributed to heterogeneous sarcomere elongation during eccentric contractions, leading to the so-called “popping sarcomere” phenomenon and subsequent reduction in force-generating capacity.^[94]^ Additionally, damage to the T-tubules and sarcoplasmic reticulum disrupts calcium release and reuptake, impairing excitation–contraction coupling, and further reducing contractile function.^[95]^

Beyond strength, EIMD can also impair explosive power owing to diminished elastic energy storage and disruption of the stretch-shortening cycle.^[96,97]^ Endurance performance may also decline, likely due to mitochondrial dysfunction and the increased energetic cost of contracting structurally damaged fibers.^[98,99]^ Furthermore, range of motion may be restricted by intramuscular edema and inflammation.^[33]^ Taken together, these multidimensional performance decrements highlight the importance of evaluating functional outcomes along with biochemical indices.

Five RCTs assessed the effects of caffeine on performance loss following EIMD. The results were heterogeneous across the protocols and outcome measures.

MVIC was examined in 2 studies. Chen et al^[52]^ found that 6 mg/kg caffeine significantly attenuated MVIC loss both pre- and post-fatigue, whereas Maridakis et al^[54]^ observed a nonsignificant improvement with 5 mg/kg caffeine. These discrepancies may reflect differences in participant profiles (trained athletes vs untrained women) and damage-inducing protocols (downhill running vs electrically stimulated contractions). Green et al^[65]^ reported that 6 mg/kg caffeine significantly improved isokinetic torque but had no effect on isometric strength, possibly due to enhanced motor unit recruitment or modulated pain perception. In contrast, Al-Nawaiseh et al^[60]^ found no beneficial effect of 5 mg/kg caffeine on 5-km running performance, heart rate, or perceived exertion 48 hours post-damage. Caldwell et al^[51]^, assessing lower extremity function after a 164 km cycling event, observed transient improvement only on the 1st day of midday measurement.

Overall, evidence suggests that caffeine may partially mitigate performance decrements, particularly in dynamic strength and torque-based measures, yet its effects appear transient and context-dependent. Variability across studies likely stems from differences in exercise modality, participant training status, timing of supplementation, and dose–response relationships. Consequently, although caffeine may offer short-term functional benefits, its role as a consistent recovery aid following EIMD remains inconclusive.

### 4.4. Assessment of methodological quality

The methodological quality of the studies included in this review was evaluated using the PEDro scale, which is a validated tool for assessing the internal validity of RCTs. Overall, the majority of studies demonstrated high methodological quality, with 5 studies achieving maximum scores, reflecting robust design and reporting. However, 6 studies (40%) exhibited moderate quality (scores between 6 and 8), primarily due to deficiencies in blinding and follow-up criteria, which must be considered when interpreting the findings (Table 2).

A notable strength across the included literature is the predominance of double-blind crossover designs (77.3%), which reduce between-subject variability and require smaller sample sizes, thereby enhancing internal validity and cost-effectiveness. Most studies also implemented adequate washout periods (≥7 days), minimizing the potential carryover effects between interventions.

However, several methodological limitations were identified. The lack of participant blinding in 40% and practitioner blinding in 20% of the studies may have introduced expectancy bias, especially in outcomes involving subjective measures such as pain perception and fatigue. Additionally, there was substantial heterogeneity in the exercise protocols used to induce muscle damage, ranging from isokinetic and eccentric contractions to prolonged endurance events such as 164 km cycling. As different exercise modalities elicit distinct magnitudes and mechanisms of muscle damage, this heterogeneity undermines comparability and limits the feasibility of quantitative synthesis.^[79]^

Another critical methodological issue is the timing of caffeine administration. In most studies (73.3%, n = 11), caffeine was consumed prior to exercise, which may have confounded the interpretation of the outcomes. Pre-exercise supplementation can enhance exercise intensity and workload, thereby increasing the extent of muscle damage independent of its regenerative effects.^[100]^ Consequently, even if caffeine exerts no direct influence on recovery, differences in the initial damage severity between groups may lead to misleading conclusions regarding its effects on muscle regeneration.^[75,88]^

Furthermore, habitual caffeine intake, a key moderator of ergogenic response, was low in most participants (57.7%), suggesting that the observed analgesic and ergogenic effects might be overestimated in populations with habitually low caffeine intake.^[101]^ Therefore, extrapolation of these findings to athletic populations with high habitual caffeine consumption should be approached cautiously.

In summary, although the included studies generally exhibited strong methodological quality, limitations related to blinding procedures, heterogeneity in exercise protocols, and timing of supplementation remain significant challenges.

### 4.5. Limitations and future directions

This systematic review aimed to synthesize current evidence from RCTs investigating the effects of caffeine supplementation on EIMD. Nevertheless, this study has several limitations. The literature search was restricted to the PubMed, Scopus, and Google Scholar databases. Although these databases are among the most comprehensive sources in the field, the exclusion of other relevant databases, such as Web of Science, Embase, and SPORTDiscus, may have resulted in the omission of some eligible studies. Despite the inclusion of a citation-tracking strategy to broaden the scope, it remains possible that the existing literature was not fully captured.

Another important limitation arises from the fact that all included studies relied exclusively on indirect markers, because no RCTs employing direct measurement techniques, including magnetic resonance imaging or muscle biopsy, were identified. Consequently, the conclusions of this review are confined to indirect evidence and may not fully reflect the structural extent of the muscle damage. For instance, CK levels can be influenced by factors unrelated to damage severity, such as hydration status and exercise modality,^[87,88]^ while DOMS ratings may be confounded by caffeine’s known analgesic properties, potentially mediated through adenosine receptor antagonism.^[30,80]^ Therefore, the clinical and practical implications of these findings warrant cautious interpretation.

The methodological heterogeneity among the included studies also poses a challenge for interpretation. Considerable variations were observed in the exercise protocols used to induce muscle damage (e.g., resistance training, downhill running, and cycling), caffeine dosage (3–6 mg/kg), timing of administration (pre- vs post-exercise), and assessment intervals. These differences hinder direct comparisons and preclude the possibility of conducting a quantitative meta-analysis. Given the absence of a quantitative meta-analysis, formal statistical assessment of publication bias using funnel plots or Egger regression test was not feasible. Therefore, the possibility of publication bias cannot be completely excluded and should be considered when interpreting the findings. Furthermore, the participant population primarily consisted of young, healthy, and physically active individuals, including amateurs and professional athletes. Therefore, the generalizability of these findings to sedentary populations, older adults, and female athletes is limited. Most participants were low-habitual caffeine consumers, which restricts the ability to infer the potential responses of individuals with high habitual caffeine intake. In addition, inconsistencies in washout periods across crossover designs may have introduced bias, affecting the accuracy of reported outcomes.

Future studies should aim to overcome these limitations by expanding the database search strategy, incorporating direct measurement methods, and adopting standardized exercise protocols with consistent caffeine administration schemes. Including larger and more diverse populations would enhance external validity. Moreover, precise reporting of habitual caffeine intake and careful planning of washout periods are essential for reducing bias. Collectively, such methodological refinements will improve the robustness and comparability of future findings and contribute to a clearer understanding of the role of caffeine in modulating EIMD and recovery.

## 5. Conclusion

This systematic review synthesized evidence from 15 RCTs that investigated the effects of caffeine supplementation on EIMD using indirect markers. Overall, the majority of studies reported no significant differences in circulating biochemical indicators, such as CK, suggesting that caffeine neither markedly attenuates nor exacerbates muscle damage at the biochemical level. Although CK is widely used as a marker, its limited validity in reflecting functional impairment may partially explain the inconsistency in the results.

Regarding DOMS, the findings were heterogeneous. While several studies have demonstrated the pain-relieving effect of caffeine, others have reported neutral or even adverse outcomes. These discrepancies may be attributed to variations in exercise modality, dosage, timing of ingestion, and individual factors such as habitual caffeine consumption or sensitivity. Similarly, the influence of caffeine on physical performance loss following muscle damage has been inconsistent. In some studies, caffeine partially mitigated the decline in strength and power; however, this effect was not consistently observed across all performance measures.

Taken together, current evidence suggests that caffeine supplementation may offer modest and context-dependent benefits in reducing perceived muscle soreness and preserving performance following EIMD; however, it does not appear to exert a consistent effect on biochemical markers of muscle disruption. Notably, the magnitude of these benefits may vary according to individual characteristics: the analgesic effect of caffeine appears to be more pronounced in men than in women, and the ergogenic and analgesic effects reported in this review were predominantly derived from individuals with habitually low caffeine intake, which may limit the generalizability of these findings to populations with high habitual caffeine consumption. The observed variability across studies underscores the need for future research employing standardized exercise protocols, controlled dosing regimens, and direct measurement techniques to clarify the mechanistic role of caffeine in muscle damage and recovery.

From an applied perspective, caffeine supplementation may serve as a supportive strategy for alleviating post-exercise discomfort and maintaining functional capacity, particularly in low-habitual caffeine consumers. However, given the sex- and habituation-related variability identified in this review, its use should be individualized based on exercise type, timing, and tolerance, and should not be considered a definitive intervention for mitigating muscle damage.

## Acknowledgments

We thank Fahri Safa Çınarlı for his valuable feedback on this manuscript. This review received no external funding and was conducted independently under the support of Inönü University. The authors declare no conflicts of interest.

## Author contributions

**Conceptualization:** Süleyman Mercan, Armağan Şahin Kafkas, Muhammed Emin Kafkas.

**Formal analysis:** Süleyman Mercan.

**Investigation:** Süleyman Mercan, Armağan Şahin Kafkas, Ilgın Ali Coşkun, Muhammed Emin Kafkas.

**Methodology:** Süleyman Mercan, Armağan Şahin Kafkas, Ilgın Ali Coşkun, Muhammed Emin Kafkas.

**Supervision:** Muhammed Emin Kafkas.

**Writing – original draft:** Süleyman Mercan, Armağan Şahin Kafkas, Ilgın Ali Coşkun, Muhammed Emin Kafkas

**Writing – review & editing:** Süleyman Mercan, Muhammed Emin Kafkas.
